# Pixel-Reasoning-Based Robotics Fine Grasping for Novel Objects with Deep EDINet Structure

**DOI:** 10.3390/s22114283

**Published:** 2022-06-04

**Authors:** Chaoquan Shi, Chunxiao Miao, Xungao Zhong, Xunyu Zhong, Huosheng Hu, Qiang Liu

**Affiliations:** 1 School of Electrical Enginnering and Automation, Xiamen University of Technology, Xiamen 361024, China; shichaoquan@s.xmut.edu.cn (C.S.); miaochunxiao@s.xmut.edu.cn (C.M.); 2 School of Aerospace Engineering, Xiamen University, Xiamen 361005, China; zhongxunyu@xmu.edu.cn; 3 School of Computer Science and Electronic Engineering, University of Essex, Colchester CO4 3SQ, UK; hhu@essex.ac.uk; 4 Department of Psychiatry, University of Oxford, Oxford OX1 2JD, UK; qiang.liu@psych.ox.ac.uk

**Keywords:** pixel-level reasoning, robotics fine grasping, EDINet deep network

## Abstract

Robotics grasp detection has mostly used the extraction of candidate grasping rectangles; those discrete sampling methods are time-consuming and may ignore the potential best grasp synthesis. This paper proposes a new pixel-level grasping detection method on RGB-D images. Firstly, a fine grasping representation is introduced to generate the gripper configurations of parallel-jaw, which can effectively resolve the gripper approaching conflicts and improve the applicability to unknown objects in cluttered scenarios. Besides, the adaptive grasping width is used to adaptively represent the grasping attribute, which is fine for objects. Then, the encoder–decoder–inception convolution neural network (EDINet) is proposed to predict the fine grasping configuration. In our findings, EDINet uses encoder, decoder, and inception modules to improve the speed and robustness of pixel-level grasping detection. The proposed EDINet structure was evaluated on the Cornell and Jacquard dataset; our method achieves 98.9% and 96.1% test accuracy, respectively. Finally, we carried out the grasping experiment on the unknown objects, and the results show that the average success rate of our network model is 97.2% in a single object scene and 93.7% in a cluttered scene, which out-performs the state-of-the-art algorithms. In addition, EDINet completes a grasp detection pipeline within only 25 ms.

## 1. Introduction

With the development of visual sensor technology [1], robots widely use visual sensors to understand the surrounding complex environment, such as segmenting the scene into component parts, recognizing what these parts are, and eliminating ambiguity between similar objects, while the visual perception technique has always been an important research area for robot grasping behaviors development [2,3,4,5]. 

Grasping is a necessary ability for human beings; so long as we look at objects and pay attention to the surrounding environment, people can easily make the best grasping posture according to the shape and size of the object and pick it up accurately. However, how to enable the robot to make accurate and collision-free reliable grasping is still challenging: the grasping representation and grasping reasoning problems for the physical attributes of the gripper and the network algorithm are still open problems that are worth exploring.

For robotic grasping representation, a complete gripper configuration should include a 6D grasping pose and grasping width [6]. Thus, it is very complicated to reason all possible grasping configurations. In order to facilitate the different robotic grasping tasks, the grasping representation is mapped into the two-dimensional image plane [7,8,9]. In actual tasks, this mapping method often sets the size of the gripper to a constant or fixed value, and the size of the gripper is not potentially related to the size of the objects. Generally, most methods maximize the gripper jaw opening and then close the gripper directly. Thus, these grasping methods will cause the gripper to collide with other objects in a small space, which can lead to failed grasping.

In the grasping reasoning, the network algorithm outputs the sum of all the gripper configurations of the object that can be grasped by the robotics. In the previous work, such as based on the rectangular representation method, multiple discrete grasping candidates are predicted from RGB or RGB-D images, but it resulted in a time-consuming process for grasping detection. The improved methods try to shorten the computation time by preprocessing the candidate rectangle or synchronously extracting the candidate rectangle and the prediction confidence. However, this method will ignore the potential grasping pose [10,11,12].

In order to overcome the above problems, we propose a pixel-level grasping reasoning method to generate gripper configurations on each pixel, and an encoder–decoder–inception network (EDINet) is also proposed for feature extraction and producing fine gripper configurations and grasping poses. Firstly, a new fine grasping configuration with an adaptive width for a robotic arm approaches the object to avoid the failed grasping caused by colliding with the surrounding objects. Secondly, an EDINet model generates fine grasping configurations on each pixel of grasping area. Pixel-level grasping mapping avoids omitting the ground truth grasping pose and overcomes the limitations of the current deep learning grasping methods, including time-consuming reasoning and discrete sampling of grasping candidates. Our EDINet model can effectively extract multi-scale features of objects and has good feature extraction ability for different shapes and sizes of objects. On the Cornell grasp dataset, 98.9% and 97.7% accuracy are obtained in image-wise and object-wise splitting, respectively. In the actual grasping experiment, our method achieves a 97.2% success rate in single-object scenes and a 93.7% success rate in cluttered scenes. On the desktop computer equipped with GPU, it only takes 25 ms for the network to complete a grasp detection pipeline, which meets the needs of real-time performance. 

The main contributions of our work can be summarized as follows:

- We propose a fine grasping representation model to generate the gripper configuration of parallel-jaw, which can effectively avoid the collision problem for clutter objects. Besides, the adaptive grasping width is fine for deformed or rigid objects in the grasping process;
- It is proposed to use the EDINet network to generate pixel-level gripper configurations to avoid missing potential ground truth grasp poses and reduce calculation time. The EDINet meets the real-time performance within 25 ms and achieves a very good balance in the speed and accuracy of grasping reasoning;
- Our system shows out-performance on the Cornell grasp datasets due to proper network structure, and it has been proven to be effective for novel objects in cluttered scenes. In actual robot grasping, our method has an average grasp success rate of 97.2% in a single-object scene and an average success rate of 93.7% in a cluttered scene. Moreover, our method outperforms the state-of-the-art algorithms in real application;
- Our network uses RGB-D multi-modal data to enhance the diversity and saliency of features so that it is easy to train the model and effectively improve the accuracy and success rate of grasping detection.

## 2. Related Work

### 2.1. Robotic Grasping

Many factors are involved in the actual robot grasping tasks, such as the physical properties of the objects and the robotics themselves. Thus, the grasping detection methods are roughly divided into two categories: analytical methods and empirical methods. The former uses mathematical and physical models, such as kinematics, dynamics, and geometry, to calculate stable grasping [13,14]. In previous grasping applications, these methods based on mathematics and physical models played an important role in solving the grasping problem. These methods involve a complete three-dimensional physical model of the object to simulate the grasping operation. Meanwhile, taking various constraints into account, the objective function is established to make the grasping more stable. However, the environment faced by the robot is often unknown, and the three-dimensional reconstruction of the object cannot be obtained in advance. It is difficult to model the physical interaction between the robot arm and the object, and it cannot be well transmitted to the real tasks [15,16]. In the real world, it is easier for robots to use cameras to capture RGB images and depth images than three-dimensional modeling. The empirical method does not require the 3D model of the object. The empirical method focuses on using data-driven and network learning technologies to train a grasping model from sample data and then use the learning model to detect the grasping posture of unknown objects [9,17,18,19,20,21,22].

### 2.2. Grasping Representation

Grasping on the image plane generally includes a grasping center point, grasping angle, and grasping width. Zhang et al. [15] used a five-dimensional directional rectangle to represent the gripper configuration. Mahler et al. [23] represented the grasping configuration with a point and an angle. Li et al. [24] used a 6D grasp representation. However, in practical applications, when the end-effector of the robotic arm reaches the position of the object to be grasped, the gripper is directly closed from the opened maximum width. These methods do not take into account the surrounding space of the objects to be grasped. The maximum opening width of the gripper jaw may collide with other objects, resulting in grasping failure and minimal closing, easily breaking the deformational, thin, and plastic objects. That is not a fine grasping representation.

### 2.3. Network for Grasping

In recent years, due to its excellent feature extraction and generalization ability [22], deep learning technology can directly perform grasping detection from RGB images, depth images, and RGB-D images [9,23,24,25,26]. The neural network can efficiently calculate and stably grasp. Lenz et al. [27] used a cascade neural network to detect the grasping position in the RGB-D images. Among them, the smaller network is responsible for removing low-probability grasping locations, and the larger network can extract more features. Then, the network determines the position of the candidate rectangle to obtain the optimal grasping posture. Chu et al. [28] proposed a model based on the RPN network (region propositions network), which simultaneously predicts the grasping posture of multiple targets in RGB-D images, and achieved good detection results. Depierre et al. [29] proposed a network model with scoring function. The network model evaluates the grasp ability of a given location and introduces a new loss function that associates the grasping parameter regression with the grasping ability. Guo et al. [8] used a deep network to train the fruit dataset to detect the most exposed objects and the optimal grasping posture. However, their model has no perception of the overall environment and has certain limitations. Li et al. [30] proposed a neural network for grasping detection that treats the angle learning problem as a classification rather than a regression problem. Zhang et al. [15] proposed the oriented anchor frame mechanism, which assigns different default rotation angles to the reference rectangle, and achieved good results on the Cornell grasp datasets. Nowadays, multi-modal data are used for grasping detection. Jiang et al. [31] used RGB-D images to infer the grasp based on a two-step learning process. The first step is to reduce the space, and the second step is to calculate the optimal grasping pose. In many cases, deep networks need to process millions of parameters [32,33,34] and use sliding windows to process candidate grasping objects. These methods lead to long computational grasping time. Song et al. [13] used the single-stage grasping detection network of the region proposal network and used the oriented anchors to predict the five-dimensional rectangle grasping model. Asif et al. [35] fused the CNN structure with hierarchical features to generate grasping posture and confidence at the global, regional, and pixel levels of the image to overcome the limitations of a single model. Kumra et al. [36] proposed a deep CNN network that uses residual layers to predict robust grasping. These algorithms will lead to ignoring some potential grasps and fail to generate dense predictions, which makes it difficult to predict the grasping properties of the object.

## 3. Robot Grasp Representation

**Figure 1:**
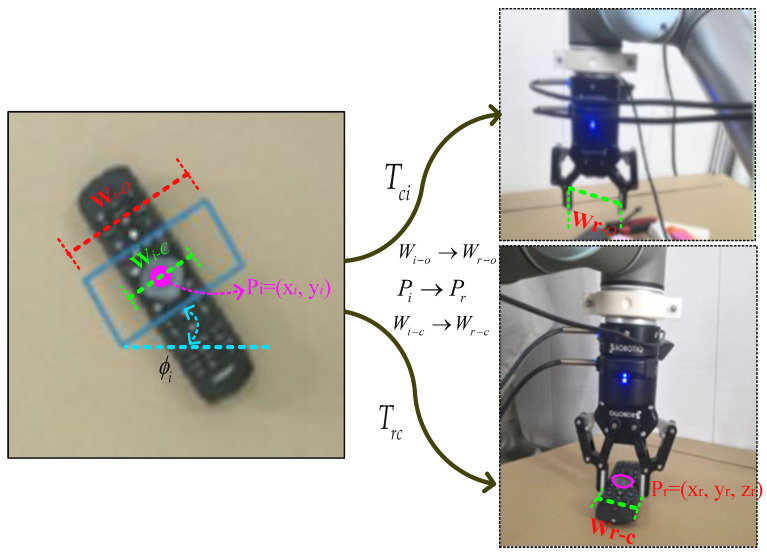
The representation of fine grasping in image and robotics workspace.

As shown in Figure 1, a fine grasping representation model with adaptive width is defined on the basis of five-dimensional grasping, as follows:

(1) $$G_{r}=(P_{r},\phi_{r},w_{r-o},w_{r-c},Q_{r})$$

where the grasping *G_r_* refers to a grasp in robot workspace, the center position $P_{r}=(x_{r},y_{r},z_{r})$ of the gripper is in the Cartesian coordinate, $\phi_{r}$ is the rotation angle around the z axis. *w_r_*_–*o*_ and *w_r_*_–*c*_ are the opening and closing width when the gripper approaches and picks up the object, respectively. Compared to the position and rotation representation alone, the increase in the gripper width allows for fine grasping performance. $Q_{r}$ is the grasp confidence for representing the success rate of grasping.

We detect a grasping representation from the RGB image $I=R^{3\times h\times w}$ and the depth image $D=R^{h\times w}$ with height *h* and width *w*, which can be defined as:

(2) $$G_{i}=\left(x_{i},y_{i},\phi_{i},w_{i-o},w_{i-c},Q_{i}\right)$$

where $P_{i}=(x_{i},y_{i})$ is the grasp center in the image coordinates, and $\phi_{i}$ is the rotation angle in the camera coordinate, which represents the rotation scalar of each point required to grasp the object of interest, and the rotation range is in $\left[-\frac{\pi }{2},\frac{\pi }{2}\right]$. $w_{i-o}$ and $w_{i-c}$ are the width of the image to be grasped at each point corresponding with gripper opening and closing. $Q_{i}$ is the grasp confidence of each point in the image, and its scalar value is between 0 and 1. The closer the value to 1, the greater the success rate of grasping. Our goal is to infer a set of grasping *G* = (*G*_1_, *G*_2_, …, *G_k_*) that maximizes the grasp success rate given a possible grasping *k*:

(3) $$\left\{G_{i}^{*}\right\}=\underset{\left|G\right|=k}{\arg\max}\sum_{G_{i}\in G}\mathrm{Pr}\mathrm{ob}\left(Q_{i}=1|I,D,G_{i}\right),$$

In order to command a robot to execute a grasp task, the pixel grasping detection should be transformed into gripper configuration. It involves system calibration and robot moving model, as follows:

(4) $$\left\{ \begin{array}{l} G_{r}=T_{rc}T_{ci}G_{i}^{*}\\ T_{rc}={\left[\begin{array}{cc} R & T\\ \overset{}{0} & 1 \end{array}\right]}^{-1}\\ T_{ci}={\left[\begin{array}{cccc} f_{x} & 0 & u_{0} & 0\\ 0 & f_{y} & v_{0} & 0\\ 0 & 0 & 1 & 0 \end{array}\right]}^{-1} \end{array}\right.$$

where *T_ci_* represents the conversion function from 2D image coordinates to camera coordinates, in which $f_{x}$ and $f_{y}$ are focal lengths and $\left(u_{0},v_{0}\right)$ are the optical center coordinates. *T_rc_* is the conversion from camera coordinates to robot workspace, in which *R* and *T* are the rotation matrix and translation matrix from the world coordinate system to the camera coordinate system, respectively.

## 4. Proposed Methods

### 4.1. The Robotics Grasping System

**Figure 2:**
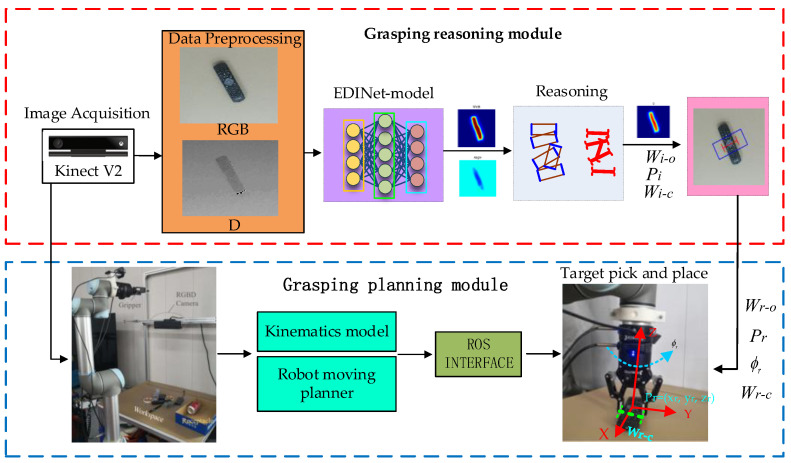
The overview of the robot grasping system.

The overview of the robotics grasping system is shown in Figure 2. It is divided into two modules: the grasping reasoning module and the grasping planning module, where the reasoning module is used to predict the appropriate grasping representation in the image space. Firstly, the RGB images are preprocessed, cropped, and resized, and the depth images are processed to remove invalid values. Subtract the mean value of the depth map and concentrate the value near 0 to maintain the depth invariance [37]. Second, the format RGB-D multi-model images are used to EDINet for grasping angle, grasping width, and appropriate grasping posture inference with the highest grasping confidence. After that, the eye-to-hand model is used to convert the grasping pose from camera coordinates to robot coordinates. Finally, the grasp planning module performs the tasks, such as execution and placement.

In our system, the grasp planning module is completed on the robot operating system (ROS). The ROS provides a related interface to connect the robot arm. It uses inverse kinematics to calculate the planned motion trajectory and then performs grasp and place actions; thus, our grasping system is suitable for most manipulator operations.

### 4.2. The EDINet Architecture

**Figure 3:**
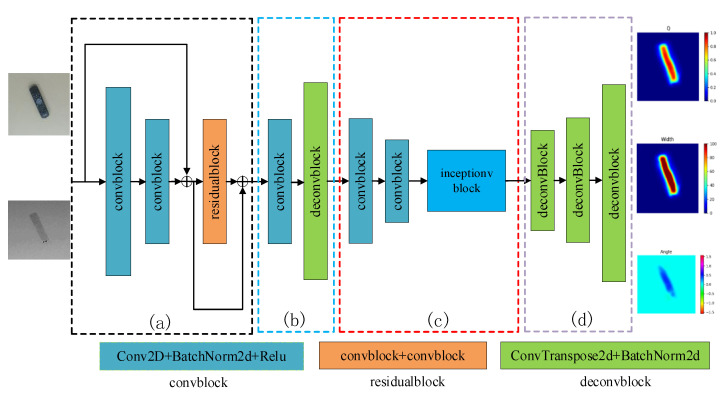
The structure of EDINet: (**a**) encoder module, (**b**) decoder module, (**c**) inception module, (**d**) up-sampling module.

In this work, the grasp detection with deep network structure is regarded as the pixel-reasoning problem. Considering the inference speed, the network should be lightweight with fewer parameters, and the networks should also use modules that reduce the number of parameters. As well as considering the accuracy of the network, the network should have a suitable depth. Thus, in Figure 3, we try to design a new EDINet network that consists of encoder module, decoder module, inception module, and up-sampling module, which is proposed to quickly generate the optimal grasping configurations.

In our EDINet architecture, as shown in Figure 3a, the encoder module consists of two convblocks and a residualblock. In convblocks, the batch normalization layer can speed up the network convergence, and the ReLU function can enhance the nonlinearity of the network. The residualblock can solve the problem of deep network degradation through identity mapping. The encoder module performs feature extraction on the inputting images. It can also extract the gripper configurations information and map it into low dimensional distribution.

In Figure 3b, we use decoder module to perform up-sampling and map the gripper configuration feature to a higher dimensional space. Considering the encoder model easily loses the spatial information of the grasp pose during the down-sampling step, we adopt a direct connection between the encoder and the decoder [37]. Bypassing the spatial information and going directly from the encoder to decoder improves accuracy and reduces processing time. In our new encoder–decoder model, the output of the encoder is used as the input of the decoder to generate the dense features of the gripper configuration. After this stage, the network completes the preliminary feature extraction and generates coarse gripper configurations.

With the increase in the number of convolutional layers, we find that the network will cause the following problems: (1) gradient vanishing, and it is difficult to optimize training the model; (2) too many parameters may also lead to over-fitting matters. These problems will cause the model to output an inaccurate gripper configuration. Thus, to solve these problems, as can be seen from Figure 3c, this paper uses inception module to increase the width and depth of the network. Considering the grasping angle, grasping width, and grasping area of different scale objects are all related to the edge information, the network uses 1 × 1, 3 × 3, and 5 × 5 convblocks to increase the receptive field, which enables the network to extract the features of the different scale objects and fuse the grasping pose information. The network also uses 1 × 1 convolutional layer in each branch to reduce the network parameters and the number of channels. In general, the inception module extracted gripper configuration features from the four branches and obtained the multi-scale features, which can effectively avoid gradient vanishing and over-fitting problems.

In Figure 3d, the up-sampling module consists of three deconvblocks; the up-sampling module used to accurately restore the grasping area due to the grasping area is smaller than object mask. The network can reason the grasp quality, grasp angle, grasp width of each pixel in the grasp region, and then the point with the maximal quality detected by network as the best grasp point. The optimal grasping model is established by using grasp point, grasp angle, and grasp width.

### 4.3. Grasping Training

In order to train the proposed model, the label data require processing, as follows:

- Grasp confidence: We regard the grasp confidence as a binary label and express it with a score between 0 and 1. The closer it is to 1, the higher the success rate of grasping.
- Grasp width: In order to achieve depth invariance, we set the grasping width $W_{i-o}$ and $W_{i-c}$ in the range of [0,$W_{max}$], and $W_{max}$ is the maximum width of the gripper. In the training process, we first scale it to [0,1] and then use the camera parameters and the measured depth to calculate the grasp width.
- Grasp Angle: Set the area of the grasp rectangle to $\delta_{t}$ and encoding the angle as a vector component on the unit circle produces a value in the range [–1,1] and eliminates the possibility of discontinuity when the angle surrounds $\pm \frac{\pi }{2}$. We use $\phi =\arctan\frac{\sin(2\delta_{t})}{\cos(2\delta_{t})}$ to represent the grasp angle.

The proposed EDINet is running on an ubuntu16.04 system with an Intel Core i9-10900K CPU and NVIDIA GeForce 3090 GPU. We use the Adam optimizer to optimize and train the network. The initial learning rate is set to 0.001. The network is trained end-to-end for 110 epochs. The learning-rate decays stepwise at rate of 0.5 times every 55 epoch.

### 4.4. Loss Function

Considering the traditional loss function $L_{2}(x)=x^{2}$ uses the square calculation, when x is greater than 1, it will magnify the error; thus, it may cause the gradient explosion problem. Further, the derivative of the loss function $L_{1}(x)=\left|x\right|$ is constant and non-derivative at 0, which may cause the model to oscillate and not be conducive to the convergence of the network, while, as shown in the Equation (5), the loss function Smooth *L*_1_ perfectly avoids the flaws of *L*_1_ and *L*_2_. Smooth *L*_1_ can limit the gradient in two ways. When the difference between the prediction value and the ground truth is too large, the gradient value will not be too large. When the difference between the prediction value and the ground truth is small, the gradient value is small enough. Based on the experiments studying the performance of above loss functions, the smooth *L*_1_ loss function is the best choice in this paper.

(5) $$Smooth_{}\;L_{1}=\{\begin{array}{l} 0.5x^{2}\begin{array}{cc} & if\;|x| \end{array}<1\\ |x|-0.5\begin{array}{cc} & otherwise \end{array} \end{array}$$

In the prediction task, the loss function of cosine grasping angle can be defined as:

(6) $$L_{\cos2\phi }=-\frac{1}{N}\sum_{i}^{N}smoooth_{L1}(\cos2\phi_{i}-\cos2{\overset{\Lambda }{\phi }}_{i}),$$

where $\cos2{\overset{}{\phi }}_{i}$ is the true value and $\cos2{\overset{\Lambda }{\phi }}_{i}$ is the predicted value. Similarly, the loss function of the sine grasping angle can be defined as:

(7) $$L_{\sin2\phi }=-\frac{1}{N}\sum_{i}^{N}smoooth_{L1}(\sin2\phi_{i}-\sin2{\overset{\Lambda }{\phi }}_{i}),$$

where $\sin2\phi_{i}$ is the true value and $\sin2{\overset{\Lambda }{\phi }}_{i}$ is the predicted value. In the prediction task, we can define the loss function of grasping width as:

(8) $$L_{w}=-\frac{1}{N}\sum_{i}^{N}smooth_{L1}(w_{i}-\overset{\Lambda }{w_{i})}$$

In the task of grasping and detecting, the total loss function can be defined as:

(9) $$L_{total}=L_{\cos2\phi }+L_{\sin2\phi }+L_{w}$$

### 4.5. Pixel-Level Grasping Detection

**Figure 4:**
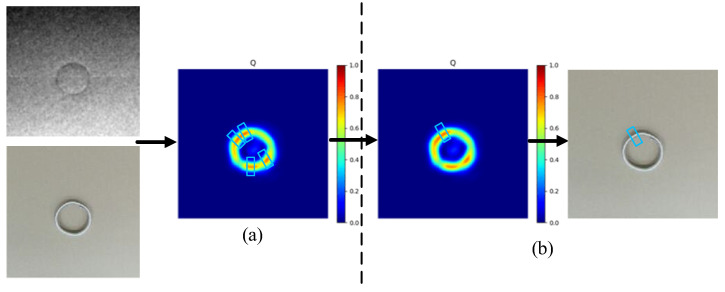
Pixel-level grasping. (**a**) Multiple grasping rectangles in multiple grasping regions, and the center of grasp rectangle is the local maximum. (**b**) The pixel point with the global maximal grasp quality is the center of the predicted grasping rectangle.

The pixel-level grasping detection method proposed in this paper is modified on the basis of Ref. [38]. In order to make the predicted grasping rectangle close to the labeled grasping rectangle, we introduce the grasping region. Firstly, the input image is initialized with all pixels being 0, and the image size is the same as the original image size. The pixels are set to 1 in the label regions, and other pixels are still set to 0. Pixels with a value of 1 constitute the grasping region and generate ground truth of grasping quality. Similarly, the same operation is performed on the grasping width and grasping angle and generates ground truth of grasping angle and grasping width. Since the length L of the labeled grasping rectangle is longer than the width of object, we select the area of length L/4 around the center as the grasp region. We take the point with the maximal grasping quality in the grasping region as the grasping point (x, y). At the same time, the grasping point with maximal grasping quality is taken as the center of the predicted grasping rectangle. The goal of the network is to make the predicted grasp rectangle close to the ground truth. An object may have multiple grasp regions. If multiple grasping rectangles need to be detected, we use the point with the maximal grasp quality in each grasping area as the grasping point to generate the corresponding grasping pose (see Figure 4a). If only a single grasping rectangle needs to be detected, the network directly searches all grasp regions and selects the pixel point with the global maximal grasp quality as the center of the predicted grasping rectangle (see Figure 4b).

## 5. Implementation Details

### 5.1. Training Dataset

Cornell and Jacquard grasp datasets are commonly used in robot grasping training, so, in this work, we use these two public datasets to train and evaluate our model. The Cornell grasp dataset contains approximately 885 RGB-D images with a resolution of 640 × 480 pixels and 240 different real objects. It includes 5110 positive sample grasps and 2909 negative sample grasps manually labeled. Our pixel-level grasping means that we should improve the dataset to provide multiple grasping labels for each image and have the most realistic estimate of the grasp map. We also augment the dataset by data augmentation (cropping, rotating, scaling) to enhance the quality of the dataset. The Jacquard grasp dataset is larger than the Cornell grasp dataset, which is based on CAD model; this grasp dataset contains 54 K RGB-D images and annotations manually marking the grasp location. The dataset has a total of more than 1 million grasp examples; thus, the Jacquard grasp dataset is large enough to train our network model without data enhancement.

### 5.2. Metrics for Grasp Detection

If the following two conditions are met, the predicted result of network is deemed to possess reliable grasping:

(1) The rotation angle difference between the predicted grasp rectangle and the ground truth rectangle is less than 30°;
(2) The Jaccard index between the predicted grasping rectangle and the ground truth rectangle is more than 0.25, where the Jacquard index is defined as:
  (10) $$J(G,\overset{\wedge }{G})=G\cap \overset{\wedge }{G}/G\cup \overset{\wedge }{G}$$

In which G is the predicted value and $\overset{\Lambda }{G}$ is the labelled grasp (ground truth).

### 5.3. Test in Datasets

We test the grasping detection accuracy and robot grasping success rate of our method on household objects. In experiments, 50 common household objects were selected. Each object is different in size and shape, and there is almost no similarity between them. Each object is tested with 10 different grasping positions and directions for a total of 500 grasp attempts. In actual work, the robot must not only grasp in an isolated environment but also grasp objects in a cluttered environment. We choose 50 unknown and novel objects and choose 15 objects from these 50 objects to randomly create a cluttered scene to test the grasping performance.

## 6. Results and Analysis

In this section, we evaluate the performance of IEDNet on the Cornell and Jacquard grasp datasets. In order to test the generalization ability of the network, we use image-wise (IW) and object-wise (OW) splitting to show the promotion ability of the model to any type of object. The IW is used to test the generalization ability of the network model when objects have different poses, and the OW is for new objects grasping.

### 6.1. Ablation Experiment on Network

In this test, we conducted an ablation experiment to assess the impact of each model for the EDINET networks. The network is evaluated on the Cornell grasp dataset with the RGB-D images. Firstly, we use the encoder–decoder module but without residualblock as the baseline. Table 1 shows the results of the network with different modules, and one can see that the baseline obtained an accuracy of 94.9% and 94.7% in IW splitting and OW splitting, respectively. The encoder–decoder module with residualblock (see Figure 3a) increases the accuracy by 1.3% and 1.2%, respectively, because the residualblock is perfect for solving the problem of deep network degradation through identity mapping. The inception module (see Figure 3c) improves the network grasping accuracy by 2.1% and 1.4% because it increases the receptive field, which enables the network to extract different scale features to fuse the grasping pose information. The up-sampling module (see Figure 3d) increases accuracy by 0.6% and 0.4% because the deconvblocks can effectively reduce the loss of detailed information during the up-sampling process. 

### 6.2. Test Results on the Cornell Grasp Dataset

The IW and OW data splits are used in accordance with the cross-validation setup, the same as the previous works [39,40]. As shown in Table 2, EDINet is compared with the state-of-the-art grasping detection algorithms; the results show that our network obtains the maximum accuracy rate with less scene information. The EDINet achieves the best accuracy of 98.9% and 97.7% in IW and OW splitting, respectively. Works such as those of Refs. [19,24,38,41,42,43,44,45,46,47,48,49,50,51,52] use grasping rectangles to represent gripper configurations without considering grasping width, and those of Refs. [35,51,53,54] use candidate grasping rectangles for sampling and sorting. These algorithms will lead to ignoring some potential grasps and fail to generate dense predictions. The grasping pose predicted by their network is a set of discrete grasping rectangles, which is inconsistent with the actual grasping attributes of the object. However, the EDINet proposed in this paper is a pixel-level network that directly generates gripper configurations on each pixel, which is more in line with the grasping properties of objects. Additionally, the works in Refs. [30,33,43,50] use discrete sampling, resulting in long computation times. However, we use EDINet to directly output the grasp pose on each pixel, which can solve time-consuming problems.

In addition, compared with other methods [34,36,39,53], our network has fewer parameters, and the grasping detection speed is faster. Asif et al. [34] use a set of up-sampling to predict the gripper configurations on the pixel, but only using the up-sampling layers cannot adapt to objects of different scales. However, our network introduces the inception module to improve the adaptability to different scales, which can improve the feature extraction ability of the network and improve the accuracy of grasping detection. For the network constructed in Ref. [38], our method is slightly slower than it, but the accuracy of our network is much higher. Thus, compared with these methods, our network achieves a good balance in terms of speed and accuracy. 

The Jaccard index in Equation (10) is important for evaluating the performance of grasp detection methods; thus, our method is also investigated under the different Jaccard indexes, and we set the Jaccard index to 0.20, 0.25, 0.30, 0.35, and 0.40, respectively, to test the network detection performance. Table 3 provides the results of different Jaccard indexes on the grasping detection accuracy based on the Cornell grasp dataset. From the results, we can see that, for IW splitting and OW splitting, the grasping detection accuracy of the proposed method decreases with the Jaccard index increasing. However, compared with other methods, our method still has high accuracy. The results also show that our method has excellent performance under different Jaccard indexes, which reflects the stable grasp detection ability for unknown objects and novel objects.

**Figure 5:**
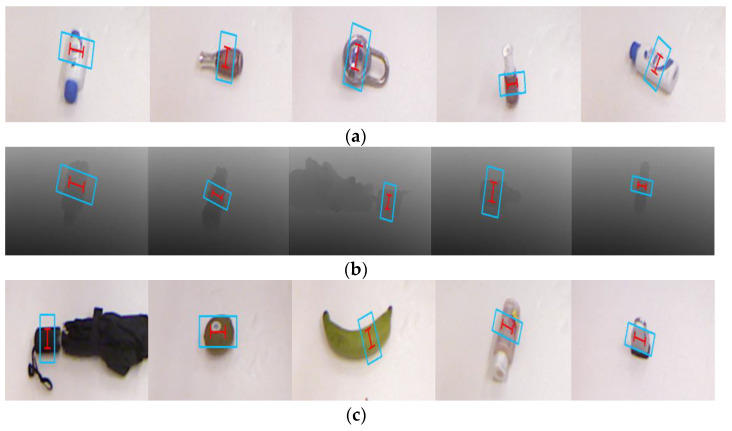
Grasping detection results on the Cornell dataset: (**a**) the evaluation results using RGB images, (**b**) the results using depth images, (**c**) the results using RGB-D image. The blue rectangle refers to the opening width when the gripper approaches the object, and the red “I” represents the closing width when the gripper picks up the object.

In Figure 5, we visualized the grasping detection results on the Cornell dataset. When the RGB images are used to train the network, an accuracy of 97.8% is obtained in IW splitting and an accuracy of 96.6% is obtained in OW splitting. When only using the depth images to train the network, the EDINet can achieve an accuracy of 95.5% in IW splitting and an accuracy of 93.2% in OW splitting. When the RGB-D multimodal data are used as the training input, our EDINet module achieves accuracies of 98.9% and 97.7% in IW and OW splitting, respectively. Experiments show that the RGB-D multimodal dataset is conducive to the network reasoning performance, which can improve the grasping detection accuracy. The Cornell dataset has been enhanced to improve the overall performance of the EDINet network. The term grasp detection pipeline is often used as a measure of grasp detection speed [38,43,51]. The EDINet only completes a grasp detection pipeline within 25 ms, which means that the inference speed of our network reaches 1/0.025 s = 40 fps, which can meet real-time applications.

### 6.3. Test Results on the Jacquard Dataset

**Figure 6:**
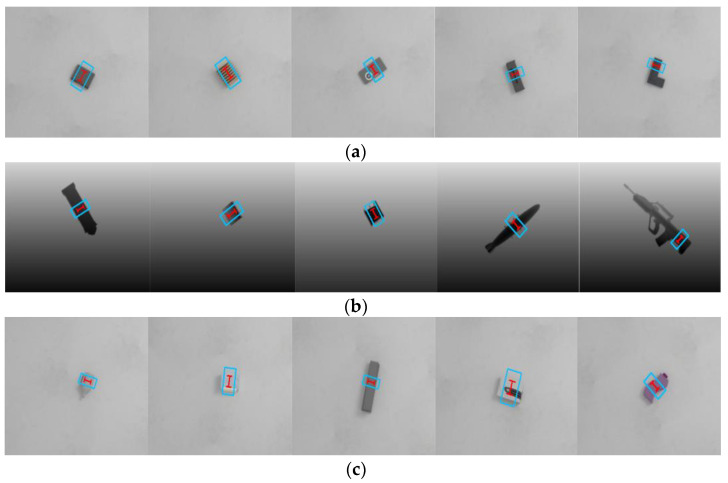
Grasping detection results on the Jacquard grasp dataset: (**a**) the results using RGB images, (**b**) the results using depth images, (**c**) the results using RGB-D images. The blue rectangle refers to the opening width when the gripper approaches the object, and the red “I” represents the closing width when the gripper picks up the object.

Since the Jacquard grasp dataset is much larger than the Cornell grasp dataset, in this test, we do not use OW splitting and data enhancement. We trained on 80% of the Jacquard grasp dataset and validated on the remaining 20%. In order to verify the superiority of our algorithm, we compared with the state-of-the-art algorithms on the Jacquard grasp dataset. The results are shown in Table 4, and Figure 6 shows the visualized grasping detection results. When only RGB images are used, the EDINet achieves an accuracy of 95.5%, and, when only the depth images are used for the network, our model obtains an accuracy of 94.9%; when RGB-D images are used to test our network, the method achieves the best grasping detection accuracy of 96.1%; thus, our method outperforms the state-of-the-art algorithms. Since the depth image can provide spatial information of objects and our network uses RGB-D multi-modal data, which can enhance the diversity and saliency of features, it is effective improve the detection accuracy and success rate of grasping.

## 7. Robot Fine Grasping

### 7.1. Adaptive Closing Width Test

**Figure 7:**
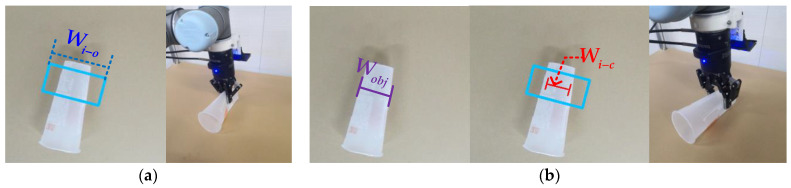
Robot close grasping test results. (**a**) The conventional grasping method directly closing; it easily broke the objects; (**b**) our grasping method with adaptive closing width, which is fine for objects. The blue rectangle refers to the opening width when the gripper approaches the object, and the red “I” represents the closing width when the gripper picks up the object.

In conventional grasping methods, after the grasping configurations are generated, the gripper will be closed directly (set the gripper to “close” in the code) [16,27,38]. As shown in Figure 7a, when using these methods to grasp thin or fragile objects, the properties of the objects are often destroyed to a large extent.

In this paper, we proposed the adaptive closing width $\left(W_{i-c}\right)$. When robot grasped an object, in the code program command, we did not choose to fully close the gripper jaws but to make the gripper jaws close to $W_{i-c}$. Here, setting up $W_{i-c}$ is mainly used for grasping thin or fragile objects. As shown in Figure 7b, we define that the width of the object as $W_{obj}$. When $W_{i-c}$ must be less than $W_{obj}$, the object can be grasped successfully. Since $W_{i-c}$ closely related to $W_{i-o}$, we define $W_{i-c}$ = μ$W_{i-o}$. We tested five cases of μ=0.1,0.2,0.3, 0.4, and 0.5, respectively. In each case, the robotic arm grasped the objects 100 times. In the three cases of μ = 0.1, 0.2, 0.3, due to the large degree of closure of the gripper jaws, it is easy to damage the objects when grasping thin plastic, paper cups, and other objects. When μ = 0.5, the robotic arm grasping heavier objects may cause unstable grasping and the objects may fall off. When μ = 0.4, the gripper jaw will be closed to the appropriate width, which will achieve great results in actual grasping. As Figure 7b shows, when robot grasped an object, the opening degree of the gripper changes from $W_{i-o}$ to $W_{i-c}$, so the damage to the objects will be minimized. When grasping a rigid object, it is difficult to deform the object, and the actual closing degree of the gripper is the $W_{obj}$.

### 7.2. Grasping with Adaptive Opening Test

**Figure 8:**
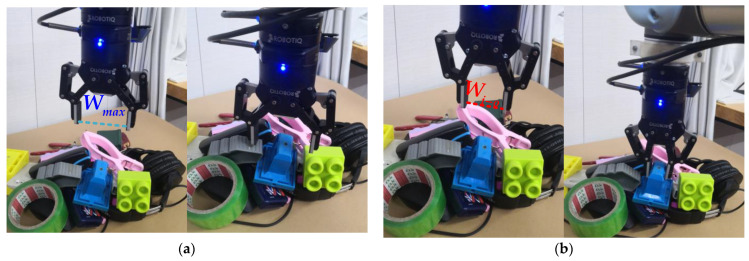
Robot open grasping results. (**a**) Robot failed grasping by the conventional method due to colliding with other objects; (**b**) robot successful grasping by our method with adaptive opening gripper configurations.

Most existing grasping methods set the opening of the gripper to the empirical value [30,31], but the gripper has no potential relationship with the size of the objects, such as the authors of Ref. [20] using a point and angle to represent the configuration of the gripper. This method sets the grasping width to a constant. As Figure 8a shows, in actual grasping, these methods are likely to collide with other objects when the gripper approaches and picks up the object. To solve this problem, we propose an adaptive opening width $W_{i-o}$, which generates adaptive grasping configurations according to the grasping attributes of the object. As Figure 8b shows, in the actual grasping experiment, the manipulator moves to about 5 cm above the object to be grasped and adjusts the grasping configurations with adaptive grasping width $W_{i-o}$.

## 8. Unknown Objects Grasping

### 8.1. Single Target Grasping Test

**Figure 9:**
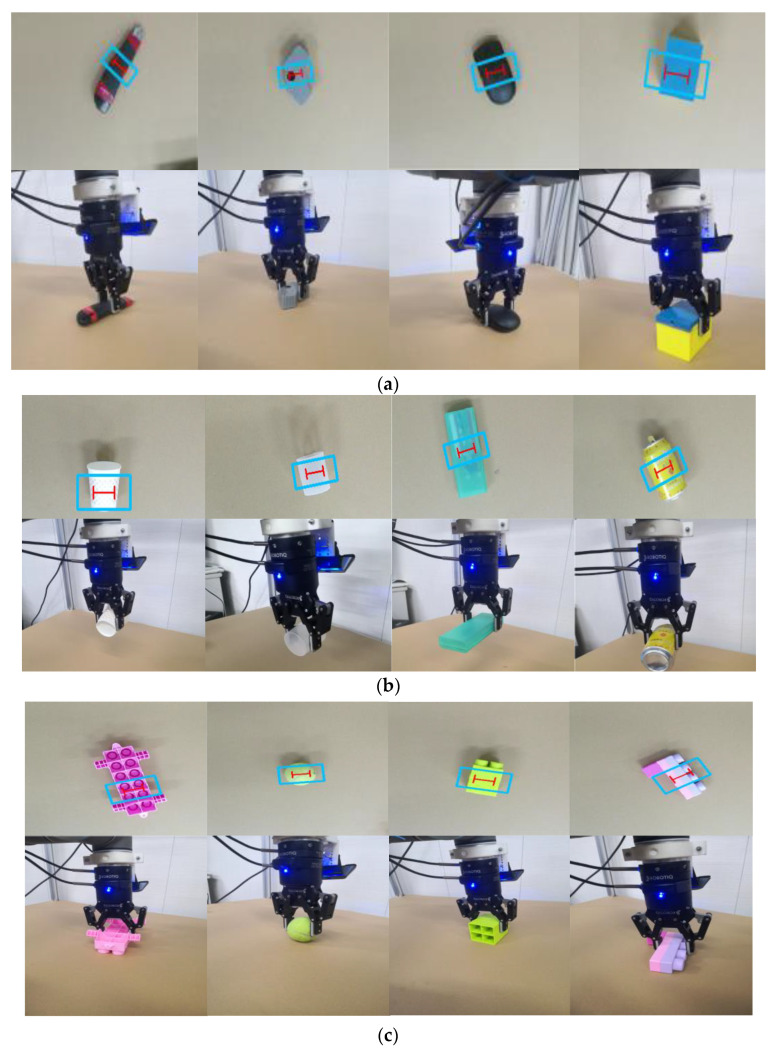
Robot grasping experiment on unknown objects: (**a**) detection and grasping on rigid objects, (**b**) robot grasping thin and easy deformed objects, (**c**) robot grasping flexible objects. The blue rectangle refers to the opening width when the gripper approaches the object, and the red “I” represents the closing width when the gripper picks up the object.

Our system has also carried out a grasping test on novel and unknown objects that do not appear in the Cornell and Jacquard grasp datasets. The grasping point with the maximal quality tends to appear in the middle of the grasping region, which makes the grasping stable. We used 50 household objects to evaluate the grasping performance of our robot arm. Each object was placed in 10 different positions. A total of 500 grasps of these objects were performed, and the robot completed 486 successful grasps, with a success rate of 97.2%. In Table 5, we compare with other works and show the performance in grasping novel objects. Besides, Figure 9 shows the detection and grasping on many objects. Based on the experimental results in Table 2 (speed) and Table 5 (accuracy), our method also achieves a very good balance in the speed and accuracy of the real-world grasping task, which is superior to the other state-of-the-art methods.

### 8.2. Cluttered Grasping Test

**Figure 10:**
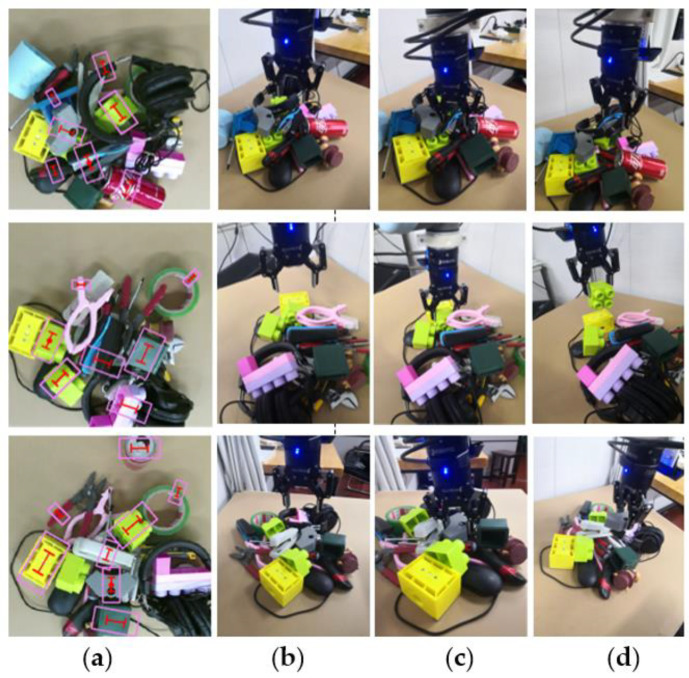
Robot grasping in different cluttered scenarios: (**a**) objects detection, (**b**) adaptive gripper configurations and robot approaching objects, (**c**) robot grasping the object, (**d**) robot picking up the object.

We further tested our method’s grasp ability in cluttered scenarios. Figure 10 shows the robot-grasped objects in different cluttered environments. In each experiment, we randomly selected 15 objects from 50 household objects. We shake the 15 test objects placed in the box and then dump them in the robot workspace. The robot grasps multiple times until the objects are cleared. We performed a total of 300 grasps in 20 experiments; the grasping success rate reached 93.7% (281/300), while we adopt the grasping configuration the same as in Ref. [34], and, regardless of grasping width, the grasping success rate only reached 88% (264/300). The performance of different algorithms in grasping unknown objects in a cluttered environment is also compared in Table 6. Compared with other methods, our method has a higher grasping success rate in cluttered scenes. In the actual grasping task, the works [34,54,58] do not consider the influence of grasping width on surrounding objects, which is likely to cause grasping failure due to collision problems (Figure 8a). Additionally, the works [38,57] do not take into account the adaptive grasping closing width, which is easy to damage the objects when grasping thin plastic, flexible, and other objects. On the contrary, according to the grasping properties of the object, our network generates adaptive gripper configurations on the pixels. The adaptive grasping width effectively avoids collisions during the grasping process. Experiments have proved that our grasping method is better than other state-of-the-art methods; our network can be generalized to all types of targets and can perform stable grasping.

Discussion: When evaluated on the same dataset, our network is superior to the state-of-the-art methods [17,24,38,39,45,46,47,52], which achieve higher grasping detection accuracy. Our network can perform pixel-level inference and prediction, which generates adaptive gripper configurations. In actual grasping tasks, the pixel-reasoning and robotic fine grasping representation proposed in this work can effectively solve the collision problem in the grasping process, thereby enabling the robot to effectively avoid grasping failure. Compared with other state-of-the-art grasping methods [47,57,58], our method has a higher success rate of grasping, and our method is comparable in grasping detection speed.

**Figure 11:**
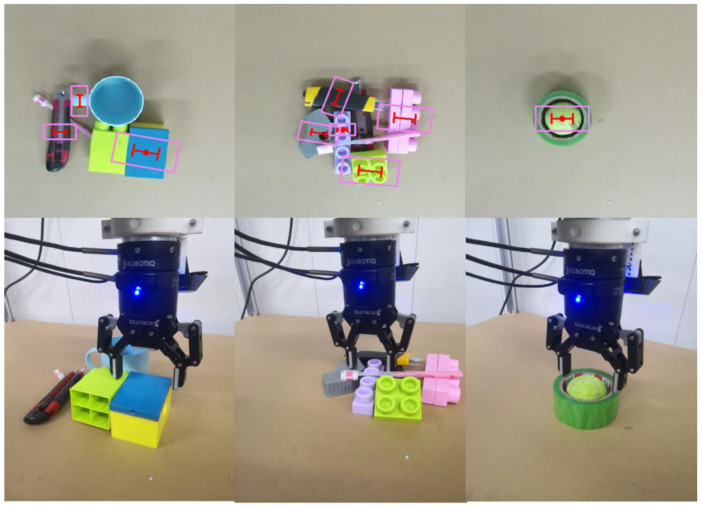
Examples of failed grasping; the most common failed grasping is that the gripper is blocked by other objects.

In the experiment, there are two main types of failed grasping: (1) when approaching an object, the gripper is blocked by other objects and objects do not have enough space for the parallel-jaw gripper (see Figure 11). (2) The object falls while being lifted.

## 9. Conclusions

In this paper, a pixel-level grasping detection method on RGB-D images was proposed. Firstly, a fine grasping representation was introduced to generate the gripper configuration of the parallel-jaw, which can effectively resolve the gripper approaching conflicts and improve the applicability to clutter objects. Besides, the adaptive grasping width is used to adaptively represent the grasping attributes, which are fine for objects that are grasped. Then, the EDINet structure is proposed to predict the fine grasping model, and it is evaluated on the public grasp dataset. Pixel-level mapping avoids the lack of a ground truth grasping pose. It also avoids the time-consuming calculation and discrete sampling of the grasping candidate rectangles, which effectively solves the limitations of the current deep learning grasping technology. The experiments show that our method outperforms other state-of-the-art algorithms in grasping detection for unknown objects in a single object scene or cluttered scene.

## Figures and Tables

**Table 1:** Ablation experiment results on EDINet.

Baseline	Encoder–Decoder	Inception Module	Up-Sampling Module	IW (%)	OW (%)
**√**				94.9	94.7
**√**	**√**			96.2	95.9
**√**	**√**	**√**		98.3	97.3
**√**	**√**	**√**	**√**	98.9	97.7

**Table 2:** Evaluation results on Cornell grasp dataset.

Authors	Algorithm	Accuracy (%)		Speed (ms)
		IW	OW	
Wang et al. [21]	DDNet	96.1	95.5	
Yu et al. [22]	TsGNet	93.13	92.99	
Yu et al. [26]	SE-ResUNet	98.2	97.1	25
Park et al. [32]	DNNs	97.7	96.1	7
Song et al. [13]	RPN	96.2	95.6	
Asif et al. [35]	DGDG	97.5		111
Kumra et al. [36]	ResNet-50x2	89.2	88.9	103
Morrison et al. [38]	GG-CNN	73	69	19
Ainetter et al. [39]	Det_Seg_refine	98.2		32
Cao et al. [41]	RSEN	96.4	-	-
Chen et al. [42]	FCN	82.8	81.9	
Zhou et al. [43]	FCGN, Resnet101	97.7	96.6	117
Shao et al. [44]	SAE+BN+SAE	95.51	-	-
Depierre et al. [45]	Grasp Regression	95.2	-	-
Yu et al. [46]	Multilevel CNNs	95.8	96.2	-
Liu et al. [47]	Mask-RCNN Q-Net, Y-Net	95.2	-	-
Redom et al. [48]	AlexNet	88.0	87.1	76
Asif et al. [49]	GraspNet	90.2	90.6	24
Guo et al. [50]	ZF-net	93.2	89.1	-
Karaoguz et al. [51]	GPRN	88.7	-	200
Kumra et al. [52]	GR-ConvNet	97.7	96.6	20
Chu et al. [53]	FasterRcnn	96.0	96.1	120
Zhang et al. [54]	ROI-GD	93.6	93.5	40
Ours	EDINet-RGB	97.8	96.6	24
	EDINet-D	95.5	93.2	24
	EDINet-RGBD	98.9	97.7	25

**Table 3:** Grasp detection accuracy on Cornell dataset with different Jaccard indexes.

Authors	Splitting	Jaccard Index				
		0.20	0.25	0.30	0.35	0.40
Song et al. [13]	IW (%)	-	95.6	94.9	91.2	87.6
Chu et al. [28]		-	96.0	94.9	92.1	84.7
Zhou et al. [43]		98.31	97.74	96.61	95.48	-
Ours		99.1	98.9	98.2	97.2	96.7
Song et al. [13]	OW (%)	-	97.1	97.1	96.4	93.4
Chu et al. [28]			96.1	92.7	87.6	82.6
Zhou et al. [43]		97.74	96.61	93.78	91.53	-
Ours		98.9	97.7	97.6	97.1	96.5

**Table 4:** Evaluation results on the Jacquard grasp dataset.

Authors	Algorithm	Accuracy (%)
Song et al. [13]	RPN	91.5
Yu et al. [26]	ResUNet	95.7
Ainetter et al. [39]	Det_Seg_refine	94.86
Liu et al. [47]	Mask-RCNN Q-Net, Y-Net	92.1
Depierre et al. [45]	Grasping Regression	85.74
Morrison et al. [38]	GG-CNN2	84
Kumra et al. [52]	GR-ConvNet	94.6
Depierre et al. [55]	AlexNet	74.2
Ours	EDINet-RGB	95.5
	EDINet-D	94.9
	EDINet-RGBD	96.1

**Table 5:** Results on single objects.

Authors	Household Objects
	Accuracy (%)
Li et al. [12]	92
Lilai et al. [20]	91.5
Yu et al. [22]	90
Morrison et al. [38]	92
Yu et al. [46]	95.82
Liu et al. [47]	94.6
Kumra et al. [52]	95.4
Chen et al. [56]	93.5
Sun et al. [57]	75.2
Ours	97.2

**Table 6:** Results in cluttered scenarios.

Authors	Objects in Clutter	
	Accuracy (%)	Adaptive Grasping Width
Yu et al. [22]	90	NO
Asif et al. [35]	90	NO
Morrison et al. [38]	87	NO
Liu et al. [47]	90.2	NO
Zhang et al. [54]	87	NO
Sun et al. [57]	75.2	NO
Li et al. [58]	87	NO
Ours	88	NO
	93.7	YES

## Data Availability

Not applicable.
